# Seroprevalence and Parasite Rates of *Plasmodium malariae* in a High Malaria Transmission Setting of Southern Nigeria

**DOI:** 10.4269/ajtmh.20-0593

**Published:** 2020-10-26

**Authors:** Eniyou C. Oriero, Adeola Y. Olukosi, Olabisi A. Oduwole, Abdoulaye Djimde, Umberto D’Alessandro, Martin M. Meremikwu, Alfred Amambua-Ngwa

**Affiliations:** 1 Medical Research Council Unit The Gambia at LSHTM, Banjul, The Gambia;; 2 Nigerian Institute of Medical Research (NIMR), Lagos, Nigeria;; 3 Calabar Institute of Tropical Disease Research and Prevention, University of Calabar Teaching Hospital, Calabar, Nigeria;; 4 Department of Epidemiology of Parasitic Diseases, Malaria Research and Training Center, University of Science, Techniques and Technology of Bamako, Bamako, Mali

## Abstract

Although *Plasmodium falciparum* continues to be the main target for malaria elimination, other *Plasmodium* species persist in Africa. Their clinical diagnosis is uncommon, whereas rapid diagnostic tests (RDTs), the most widely used malaria diagnostic tools, are only able to distinguish between *P. falciparum* and non-*falciparum* species, the latter as “pan-species.” Blood samples from health facilities were collected in southern Nigeria (Lagos and Calabar) in 2017 (October–December) and Calabar only in 2018 (October–November), and analyzed by several methods, namely, microscopy, quantitative real-time PCR (qPCR), and peptide serology targeting candidate antigens (*Plasmodium malariae* apical membrane antigen, *P*. *malariae* lactose dehydrogenase, and *P*. *malariae* circumsporozoite surface protein). Both microscopy and qPCR diagnostic approaches detected comparable proportions (∼80%) of all RDT-positive samples infected with the dominant *P. falciparum* malaria parasite. However, higher proportions of non-*falciparum* species were detected by qPCR than microscopy, 10% against 3% infections for *P. malariae* and 3% against 0% for *Plasmodium ovale*, respectively. No *Plasmodium vivax* infection was detected. Infection rates for *P. malariae* varied between age-groups, with the highest rates in individuals aged > 5 years. *Plasmodium malariae*–specific seroprevalence rates fluctuated in those aged < 10 years but generally reached the peak around 20 years of age for all peptides. The heterogeneity and rates of these non-falciparum species call for increased specific diagnosis and targeting by elimination strategies.

## INTRODUCTION

Current global efforts toward malaria elimination target the dominant malaria parasites, *Plasmodium falciparum* and *Plasmodium vivax*.^1^ However, there are three other malaria species infecting humans, with *Plasmodium malariae* being the most common worldwide, including sub-Saharan Africa (sSA).^2^ *Plasmodium malariae* causes the so-called quartan malaria, as the onset of fever occurs in an interval of 3–4 days, because of its 72-hour blood stage life cycle.^3,4^ Compared with *falciparum* malaria, it is a relatively benign infection, possibly due to low parasite densities, although it can cause severe anemia^5^ and nephrotic syndrome in both children and adults.^6,7^ *Plasmodium malariae* is often found in coinfections with other *Plasmodium* species,^3^ and because of this and the focus on *P. falciparum*, its biology, prevalence, and specific public health impact have mostly been understudied. Previous studies in areas of seasonal transmission, such as the Gambia, have reported *P. malariae* to be responsible for about half of the malaria episodes observed outside the transmission season.^8^ The implications of this dynamics are poorly understood in terms of sustained transmission, acquisition of antimalarial drug resistance, and malaria elimination.

Species-specific diagnosis of *P. malariae* is uncommon in routine malaria diagnosis; rapid diagnostic tests (RDTs), the most widely used diagnostic tools, predominantly target *P*. *falciparum*, whereas other *Plasmodium* species are identified as “pan-species.”^9^ As a result, its burden and the population at risk in most endemic areas remain largely unknown. A recent study reported persistent transmission of *P. malariae* over a 22-year period in Tanzania, suggesting that the decline in *P. falciparum* prevalence could provide a favorable ecological niche for other malaria parasite species.^10^ This has also recently been reported for *Plasmodium knowlesi*, a zoonotic malaria species, in Malaysia.^11^

There were an estimated 228 million cases of malaria worldwide in 2018, 93% in Africa, with Nigeria contributing approximately 25% of these.^1^ Malaria transmission in Nigeria is perennial, mostly due to conducive geographic landscape, high temperatures, and rainfall; about 85% of the population lives in areas of mesoendemic transmission.^12^ Although *P. falciparum* is the dominant species, responsible for more than 95% of cases of clinical malaria, *P. malariae* is found in 9.8% of malaria cases, mostly as mixed infection.^12^ Nevertheless, its prevalence, mostly determined by molecular methods, can be as high as 26%.^13^ Here, we report the detection of *P. malariae* infections from two sampling surveys aimed to describe the molecular and seroprevalence of this malaria parasite species in individuals attending health facilities in two geopolitical and climatic zones of southern Nigeria.

## METHODS

### Study site and *Plasmodium* spp. survey.

**Figure 1.**
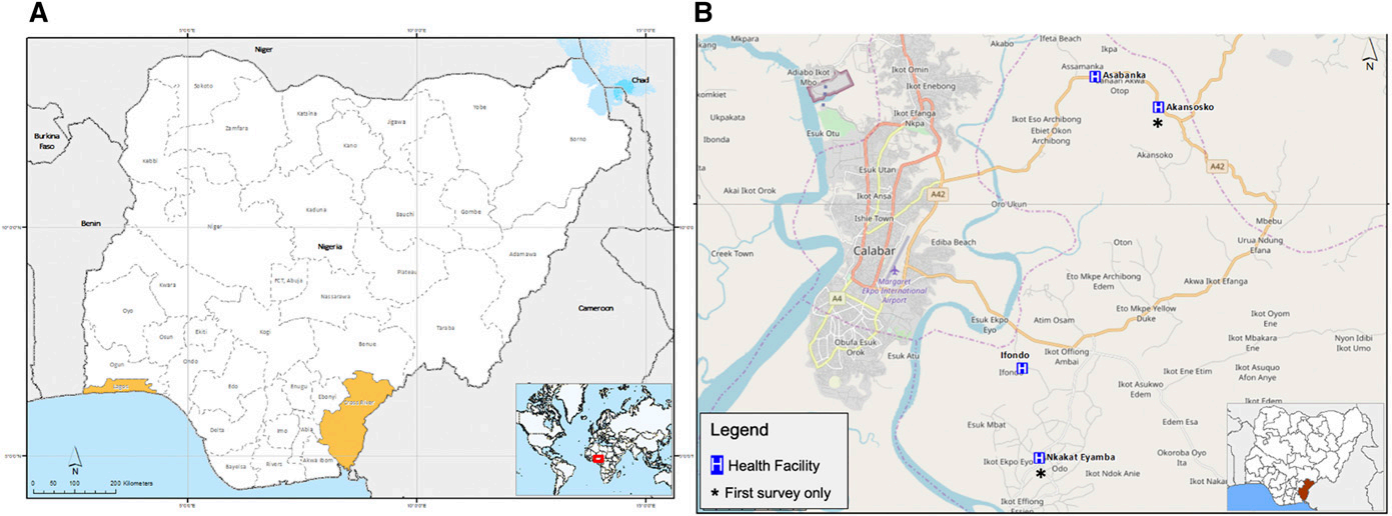
Map of study sites in Nigeria showing (**A**) Lagos (southwest zone) and Cross River (south–south zone) states; and (**B**) health facilities in Cross River state, where participants were recruited. This figure appears in color at www.ajtmh.org.

A first survey was carried out between October and December 2017 to compare *P. malariae* infections in two study sites in southern Nigeria, Lagos (Ikorodu local government area) in the southwest zone and Calabar, Cross River state (Akpabuyo local government area) in the southeast zone (Figure 1A). Suspected malaria cases sent for malaria test at the study health facilities were screened using a pan-species RDT kit, SD Bioline, Cat No 05FK60 (Abbott Diagnostics, Lake Forest, IL), after providing written informed consent to participate in the study. Blood sample was then collected from RDT-positive individuals for thick blood film preparation for microscopy and dried blood spots (DBSs) for molecular detection of *Plasmodium* species and serology assays. At the peak of the following malaria transmission season (November/December 2018), a second survey was carried out in Calabar only because of the higher prevalence of *P. malariae*, expanding to additional health facilities to increase catchment, and the total numbers screened within the sample collection period (Figure 1B). Following prior community sensitization, participants who reported at the study health facilities with suspected malaria were recruited, and samples were collected from consenting individuals as described in the first survey, irrespective of their RDT results.

Lagos is the most populous city in Africa, with a population of approximately 15 million people; it has a tropical savanna climate, with a wet season between April and October, and a dry season in the following months. The wettest month is June, with mean precipitation of 315.5 mm (12.42 in). Located near the equator, Lagos has minimal seasonal temperature variation, with high temperatures ranging between 28.3°C and 32.9°C.^14^ Calabar has a tropical monsoon climate, with a long wet season (February–November) and a short dry season covering the remaining 2 months. Temperatures are relatively constant throughout the year, with average high temperatures ranging between 25°C and 28°C; relative humidity is high with most months of the year, recording a monthly mean value of 80%, and annual average precipitation more than 3,000 mm (120 in).^15,16^

### Ethical and sample size considerations.

Ethical approval was obtained from the respective authorities in Lagos and Cross River state, references: IRB/17/038, CRSMOH/RP/REC/2017/545, and CRSMOH/RP/REC/2017/809. Written informed consent was obtained for adult participants, whereas parental or guardian consent was obtained for minors. Participants did not receive any compensation for their participation in study. All individuals diagnosed with malaria by RDTs were treated accordingly, whereas febrile individuals with a negative RDT result were referred to the health facility staff for further investigation. A minimum of 500 samples per site was required to be screened in each survey, assuming maximum malaria prevalence of 50% in suspected cases with 95% CI and 5% margin of error and an additional 30% adjustment for possible incomplete data by any of the detection methods.

### Detection of *Plasmodium* spp. parasites.

A pan-species RDT kit (SD Bioline, Cat No 05FK60), which qualitatively and differentially detects histidine-rich protein II antigen of *P. falciparum* and lactate dehydrogenase (pLDH) of *Plasmodium* species in human whole blood, was used for malaria screening according to the manufacturers’ protocol. Any sample with either P.f, pan, or both lines in addition to the control line was considered positive. Thick blood microscopy slides stained with 10% Giemsa solution were examined across 100 high-power fields by two microscopists and parasite counts recorded per number of white blood cells (WBCs) observed. In case of ≥ 20% variance in counts between primary and secondary microscopists, the counts closest to that of a third reader were retained. A slide was declared negative only after observing no parasite in 100 microscopic high-power fields, whereas parasite density was calculated using WHO protocols; the number of parasitized erythrocytes against 8,000 WBCs^17^ was expressed as parasitaemia/mL of blood.

For molecular diagnosis of *Plasmodium* spp., DNA was extracted from DBS samples using the QIAamp DNA Mini Kit (Qiagen, Hilden, Germany) according to manufacturers’ protocols. For the preliminary survey, the presence of *Plasmodium* species was detected using a commercial species-specific quantitative real-time PCR (qPCR) assay, Genesig^®^ *Plasmodium* species kits (Primerdesign™ Ltd., Chandler’s Ford, United Kingdom) according to manufacturers’ protocol. Subsequently, the presence of *P. malariae* and *Plasmodium ovale* was detected using a custom qPCR assay targeting the *Plasmepsin* gene,^18^ which showed comparable results with the Genesig kit (Primerdesign Ltd.), whereas the presence of *P. vivax* was not assessed further. Pre-amplification was performed using 0.1 μM of the forward and reverse primers for *P. malariae* (CCWR2K1_Fwd TTCAGTCAGGATATGTAAAACAAAATTATTTAGGTA; CCWR2K1_Rev CCTACTTCCCCTTCACCATAAAACA) and *P*. *ovale* (CCRR9V5_Fwd ACTCTTGGTTATTTGTCTGCACCTT; CCRR9V5_Rev CTATGTTACCATAAACAGGTTCTAAATCATCTGT), 1× Qiagen Multiplex Master Mix (QIAGEN) and 5 μL of template DNA in a 15-μL reaction. The main amplification reaction contained 3 µL of pre-amplified products in a 15-µL total reaction volume and was performed using 1× qPCR Taqman Universal Master Mix (Thermo Fischer Scientific, Waltham, MA), 0.4 μM of the forward and reverse primers, and 0.2 μM of the labeled probe (*P. malariae*, CCWR2K1_Probe TCGTCTAGTTCTATTACGTCATTTTC and *P. ovale*, CCRR9V5_Probe TCAGTTGCTTCAACAAATTT). Pre-amplification was performed under the following conditions: initial denaturation for 5 minutes at 95°C, 12 cycles of denaturation for 30 seconds at 95°C, annealing at 60°C for 1 minute, and extension for 90 seconds at 72°C, whereas main amplification and detection were performed under the following conditions: initial denaturation for 10 minutes at 95°C, 40 cycles of denaturation for 10 seconds at 95°C, and data collection for 1 minute at 60°C. *Plasmodium falciparum* was detected using a highly sensitive qPCR assay (varATS qPCR) targeting multi-copy subtelomeric sequences.^19^ Amplification reaction was performed using 1× qPCR Taqman Universal Master Mix (Thermo Fischer Scientific).

### Peptide design and serology assays.

*Plasmodium malariae*–specific peptides were designed from highly immunogenic epitopes of three *P. malariae* antigens—apical membrane antigen (AMA1), circumsporozoite surface protein (CSP), and lactose dehydrogenase (LDH) (Table 1). A *P. falciparum*–specific peptide was also designed from the same region as one of the *P. malariae* antigens – AMA1 (PmAMA1) peptides to explore species specificity. A web tool, SVMTriP (System Biology Laboratory of Chi Zhang, University of Nebraska, Lincoln, NE),^20^ was used to predict linear antigenic epitopes from the whole *P*. *malariae* antigen sequences, and the peptides were queried for species specificity using NCBI BLASTP 2.9.0+ (Bethesda, MD). The regions of immunogenic epitopes containing the peptides were then compared with orthologs of predicted *P. falciparum* epitopes available on the *Plasmodium* database^21^ and the Immune Epitope Database and Resource Analysis.^22^ The peptides indicating high species specificity and high immunogenicity were chosen for synthesis (thinkpeptides^®^ ProImmune Ltd., Oxford, United Kingdom) at 95% purity and 1–4 mg synthesis scale.

**Table 1:** List of *Plasmodium* spp. peptides for the serology assays

S/N	Peptide name	Peptide sequence	Sequence location	Antigen	Parasite spp.
1	PmAMA1-1	VLRKRYEEHADLMNLNDLSL	141–160	AMA1	*P*. *malariae*
2	PmAMA1-2	VKLYSLDGEKIVLPRIFISN	405–424	AMA1	*P*. *malariae*
3	PmCSP	AGNAAGNAAGNAAGNAAGNE	256–275	CSP	*P*. *malariae*
4	PmLDH-1	ALIVAAHGNKMVPLKRYITV	176–169	LDH	*P*. *malariae*
5	PmLDH-2	LGGVLDTSRLKYYISQKLNV	150–169	LDH	*P*. *malariae*
6	PfAMA1	TLDEMRHFYKDNKYVKNLDE	194–213	AMA1	*P*. *falciparum*

AMA1 = apical membrane antigen; CSP = circumsporozoite surface protein; LDH = lactose dehydrogenase; *P. falciparum* = *Plasmodium falciparum*; *P. malariae* = *Plasmodium malariae*.

Antibodies against the *Plasmodium* spp. peptides were detected by indirect ELISA on eluted filter paper samples. One disc (6 mm) per sample was punched and placed at the bottom of 96-well plates—flat bottom with low evaporation lids (Corning Inc., New York, NY). Serum from DBS was eluted following overnight (18 hours) incubation at 4°C in 150 µL of reconstitution buffer (1× phosphate buffer saline; 0.2% Tween20). The reconstituted solution was equivalent to a 1:100 dilution of whole blood, corresponding to 1:200 dilution of plasma or serum antibodies, if the blood were at 50% hematocrit.^23^ Peptides were coated on 96-well Nunc MaxiSorp™ Flat-Bottom Plates (Thermo Fischer Scientific) at a concentration of 3 µg/mL. Following blocking of all remaining binding surfaces with 1% skimmed milk, DBS eluate was added at a final concentration of 1:2,000 with respect to the corresponding plasma sample and analyzed in duplicate wells per sample. Each ELISA plate included a positive control, which was a pool of DBS eluate from samples confirmed to be *P. malariae* positive by microscopy and qPCR. Also included in each plate were plate blanks (no serum) and negative control, which was unexposed European pooled serum.^24^ Secondary detection was with antibody peroxidase conjugated human IgG (H&L) (Sigma-Aldrich, Taufkirchen, Germany), developed with Pierce™ TMB Substrate Kit (Thermo Fischer Scientific). The reaction was stopped using 50 μL of Stop Solution for TMB substrates (Thermo Fischer Scientific). Optical densities (ODs) were read on the EMax^®^ Plus microplate reader (Molecular Devices, San Jose, CA) at 450 nm.

### Data analysis.

Data analysis was conducted using Microsoft Excel spreadsheet, GraphPad Prism version 8.4.2 (San Diego, CA), and Stata IC 16 (Stata Corp., College Station, TX). The prevalence of *P. malariae* and non-*falciparum* species detected by the different detection methods was compared from the different study sites. Analytical procedures for reducing between-plate variation in the serology included calculating the differences between the mean ODs averaged across the duplicates of the pooled positive control for each plate and the overall mean OD values across all plates, and obtaining the estimated plate effect for a specific plate by averaging the calculated differences across all pooled controls. Test samples on a given plate were then adjusted by subtracting the estimated plate effect from their OD values.^25^ Positivity for antimalarial peptide response was obtained by modeling their normalized fold over the negative control (FOC) values (log_2_ transformed), using finite mixture models, and Gaussian distribution with two components.^23,24^ The estimated mean of the narrower distribution was used as the mean for negative samples. The cutoff for seropositivity was obtained by taking the difference between the peptide FOC values and the mean of the narrower distribution plus 2 SDs. Seroprevalence rates were analyzed according to the following age-groups: 10–20 years, 20–30 years, and > 30 years, which corresponded with estimated the duration of exposure to malaria. A three exponent nonlinear regression model and line of best fit, using the quadratic estimation, was used to obtain the predicted probability of being seropositive for a particular peptide.

## RESULTS

### Summary of participants recruited.

**Figure 2.**
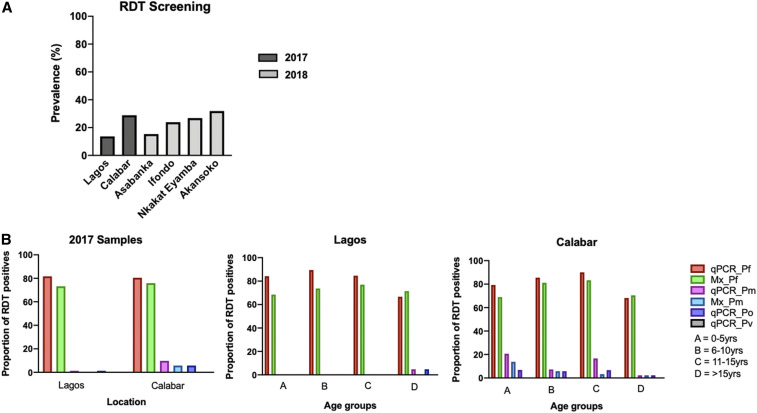
Summary of diagnostic results. (**A**) Prevalence of malaria in both surveys by RDTs. (**B**) Proportion of *Plasmodium* species detected by qPCR and microscopy in RDT-positive samples from the first survey. Mx_Pf = *P. falciparum* detected by microscopy; Mx_Pm = *P. malariae* detected by microscopy; qPCR = quantitative real-time PCR; qPCR_Pf = *P. falciparum* detected by qPCR; qPCR_Pm = *P. malariae* detected by qPCR; qPCR_Po = *P. ovale* detected by qPCR; qPCR_Pv = *P. vivax* detected by qPCR. RDT = rapid diagnostic test. This figure appears in color at www.ajtmh.org.

In 2017, a total of 243 (21.7%) individuals with a positive malaria RDT result were recruited of 1,115 participants screened—520 screened from Lagos and 595 from Calabar. Rapid diagnostic test positivity rate in Lagos was 13.7% and 28.9% in Calabar (Figure 2A). Most of the RDT-positive cases were in Calabar (71%, 172/243); children aged 6–10 years were the largest age-group (36%, 88/243; Table 2). The following year, a total of 798 participants were recruited from four health facilities in Calabar (77%, 613/798), mostly from two of the facilities (Table 2). The age distribution of the participants was different from that of the preliminary survey performed in 2017; adolescents and adults represented about half of the participants (51%, 410/798), followed by children aged 11–15 years (21%, 168/798; Table 2).

**Table 2:** Summary of participants recruited in the two malaria transmission seasons

	First survey, *n* (%)			Second survey, *n* (%)				
	Lagos	Calabar	Total	Akansoko HF	Asabanka HF	Ifondo HF	Nkakat Eyamba HF	Total
Total	71	172	243	97	266	88	347	798
Gender								
Male	31 (43.7)	88 (51.2)	119 (48.9)	45 (46.4)	120 (45.1)	38 (43.2)	147 (42.4)	350 (43.9)
Female	40 (56.3)	84 (48.8)	124 (51.0)	52 (53.6)	146 (54.8)	50 (56.8)	200 (57.6)	448 (56.1)
Age-group (years)								
0–5	19 (26.7)	29 (16.8)	48 (19.7)	10 (10.3)	49 (18.4)	18 (20.5)	18 (5.2)	95 (11.9)
6–10	19 (26.7)	69 (40.1)	88 (36.2)	6 (6.2)	71 (26.7)	9 (10.2)	39 (11.2)	125 (15.6)
11–15	13 (18.3)	30 (17.4)	43 (17.6)	7 (7.2)	38 (14.3)	6 (6.8)	117 (33.7)	168 (21.1)
> 15	21 (29.5)	44 (25.5)	65 (26.7)	74 (76.3)	108 (40.6)	55 (62.5)	173 (49.8)	410 (51.4)

HF = health facility.

### Molecular prevalence of *Plasmodium* species.

**Figure 3.**
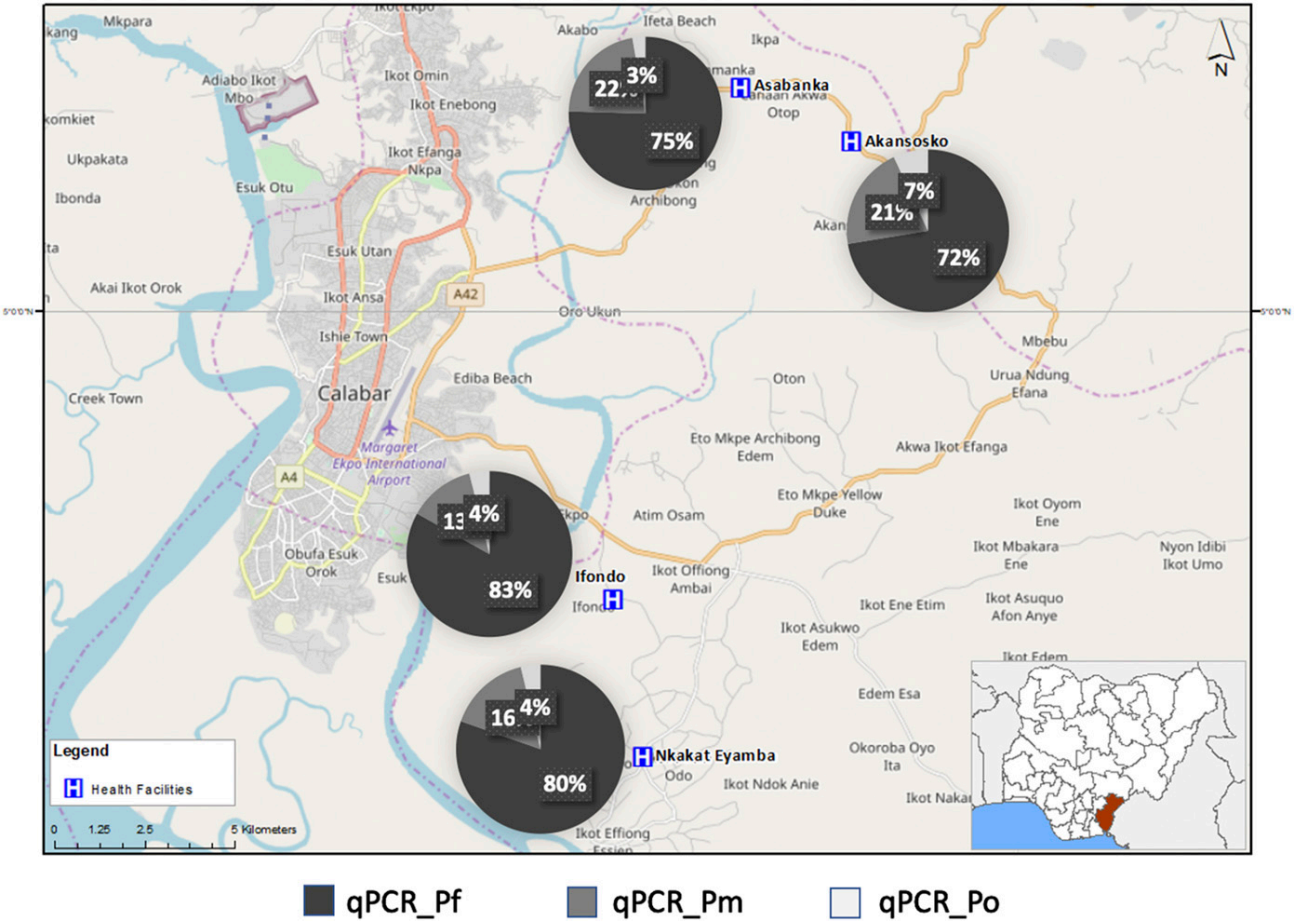
Proportion of *Plasmodium* species detected in the four villages in southeastern Nigeria, detected by quantitative real-time PCR (qPCR) (qPCR_Pf = *P. falciparum*; qPCR_Pm = *P. malariae*; qPCR_Po = *P. ovale*). This figure appears in color at www.ajtmh.org.

In 2017, when only RDT-positive individuals were recruited, the proportion of *Plasmodium* species detected in Lagos by qPCR was 81.7%, 1.4%, and 1.4% for *P. falciparum*, *P. malariae*, and *P. ovale*, respectively. In Calabar, the proportions detected were 80.8%, 9.9%, and 5.8%, respectively (Figure 2B). No *P*. *vivax* infection was detected in both locations. Similar proportions (81.7% versus 73.2% [Lagos] and 80.5% versus 75.9% [Calabar]) of *P. falciparum* infections were detected by qPCR and microscopy in the RDT-positive samples from the different study areas. However, more *P. malariae* and *P. ovale* infections were detected by qPCR, with higher proportions in Calabar than in Lagos. This emphasized the low specificity of species-specific identification of malaria parasites by microscopy. Table 3 summarizes the prevalence of *Plasmodium* spp. detected as mixed or mono infections in the four health facilities in Calabar. On average, relatively more non-*falciparum* species infections were detected in malaria samples recruited from the two facilities situated to the north of Akpabuyo local government area (26.5%) than those to the south (18.7%); however, this was not statistically significant (*P* = 0.062) (Figure 3). Despite observing a higher proportion of *P. malariae* infections in older individuals (> 15 years), the odds of having *P*. *malariae* as a mono-infection were highest in the < 5 year age-group. The least odds for mono-infections were in those of ages 11–15 years (Table 4); the odds of *P. malariae* mono infections were highest in Asabanka, with 4.5% mono-infection, and the least in Ifondo, with 1.1% mono-infection (Tables 3 and 4).

**Table 3:** Summary of non-*falciparum* species detected in south–south Nigeria as mixed and mono-infections by the different diagnostic methods—RDT, microscopy, and qPCR

RDT		*Plasmodium falciparum*, *n* (%)		Pan-spp., *n* (%)	
*Plasmodium* spp. Infection	All spp., *n* (%)	Pf_total	Pf_mono	Pan_total	Pan_only
Akansoko (97)	31 (31.9)	26 (26.8)	14 (14.4)	17 (17.5)	5 (5.2)
Asabanka (266)	41 (15.4)	40 (15.0)	35 (13.2)	6 (2.3)	1 (0.4)
Ifondo (88)	21 (23.9)	21 (23.9)	17 (19.3)	4 (4.5)	0 (0)
Nkakat Eyamba (347)	93 (26.8)	90 (25.9)	88 (25.4)	5 (1.4)	3 (0.9)

		*P. malariae*, *n* (%)		Mixed infections, *n* (%)
Microscopy	All spp., *n* (%)	Pm_total	Pm_mono	Pf + Pm
Akansoko (97)	6 (6.2)	2 (2.1)	1 (1.0)	1 (1.0)
Asabanka (266)	33 (12.4)	6 (2.3)	5 (1.9)	1 (0.4)
Ifondo (88)	15 (17.1)	0 (0)	0 (0)	0 (0)
Nkakat Eyamba (347)	99 (28.5)	16 (4.6)	15 (4.3)	1 (0.3)

		*P. malariae*, *n* (%)		*Plasmodium ovale*, *n* (%)		Mixed infections, *n* (%)			
qPCR	All spp., *n* (%)	Pm_total	Pm_mono	Po_total	Po_mono	Pf + Pm	Pf + Po	Pm + Po	Pf + Pm + Po
Akansoko (97)	25 (25.8)	6 (6.2)	2 (2.1)	2 (2.1)	1 (1.0)	3 (3.1)	0 (0)	1 (1.0)	0 (0)
Asabanka (266)	96 (36.1)	24 (9.0)	12 (4.5)	3 (1.1)	1 (0.4)	12 (4.5)	2 (0.8)	0 (0)	0 (0)
Ifondo (88)	65 (73.9)	10 (11.4)	1 (1.1)	3 (3.4)	0 (0)	8 (9.1)	2 (2.3)	0 (0)	1 (1.1)
Nkakat Eyamba (347)	224 (64.6)	42 (12.1)	9 (2.6)	10 (2.9)	2 (0.6)	32 (9.2)	7 (2.0)	0 (0)	1 (0.3)

Pf = *P. falciparum*; Pm = *P. malariae*; Po = *P. ovale*; qPCR = quantitative real-time PCR; RDT = rapid diagnostic test.

**Table 4:** Odds of *P. malariae* occurring as mono-infections or mixed infections with other *Plasmodium* spp. in the four different villages and across age-groups

	Mono infections	Mixed infections	Total	Odds*	95% CI	Odds ratio†	95% CI
Health facility							
Akansoko	2	4	6	0.5	0.09–2.73	4.5	0.25–80.57
Asabanka	12	12	24	1.0	0.45–2.23	9	0.79–101.81
Ifondo	1	9	10	0.11	0.01–0.88	1	–
Nkakat Eyamba	9	33	42	0.27	0.13–0.57	2.46	0.26–22.82
Age-group (years)							
0–5	4	3	7	1.33	0.29–5.96	16.67	1.39–199.42
6–10	3	9	12	0.33	0.09–1.23	4.17	0.55–31.75
11–15	2	25	27	0.08	0.02–0.34	1	–
> 15	15	21	36	0.71	0.37–1.39	8.93	1.58–50.32

*P. malariae* = *Plasmodium malariae*.

* Odds of *P. malariae* observed as a mono-infection compared with mixed infections.

† Odds ratio calculated relative to the least odds.

### Serological responses to the different *P. malariae* peptides.

**Figure 4.**
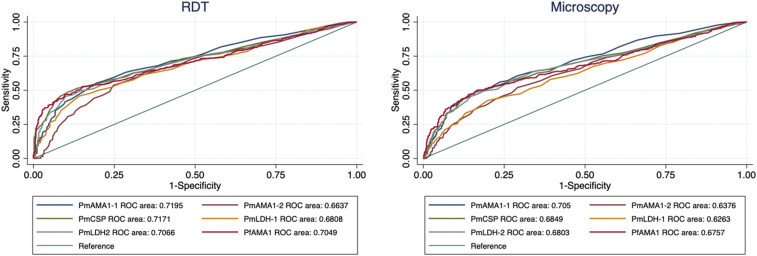
Area under the receiver operator curve (ROC) showing comparison of fold over the negative control for each of the peptides with rapid diagnostic test (RDT) and microscopy results. This figure appears in color at www.ajtmh.org.

**Figure 5.**
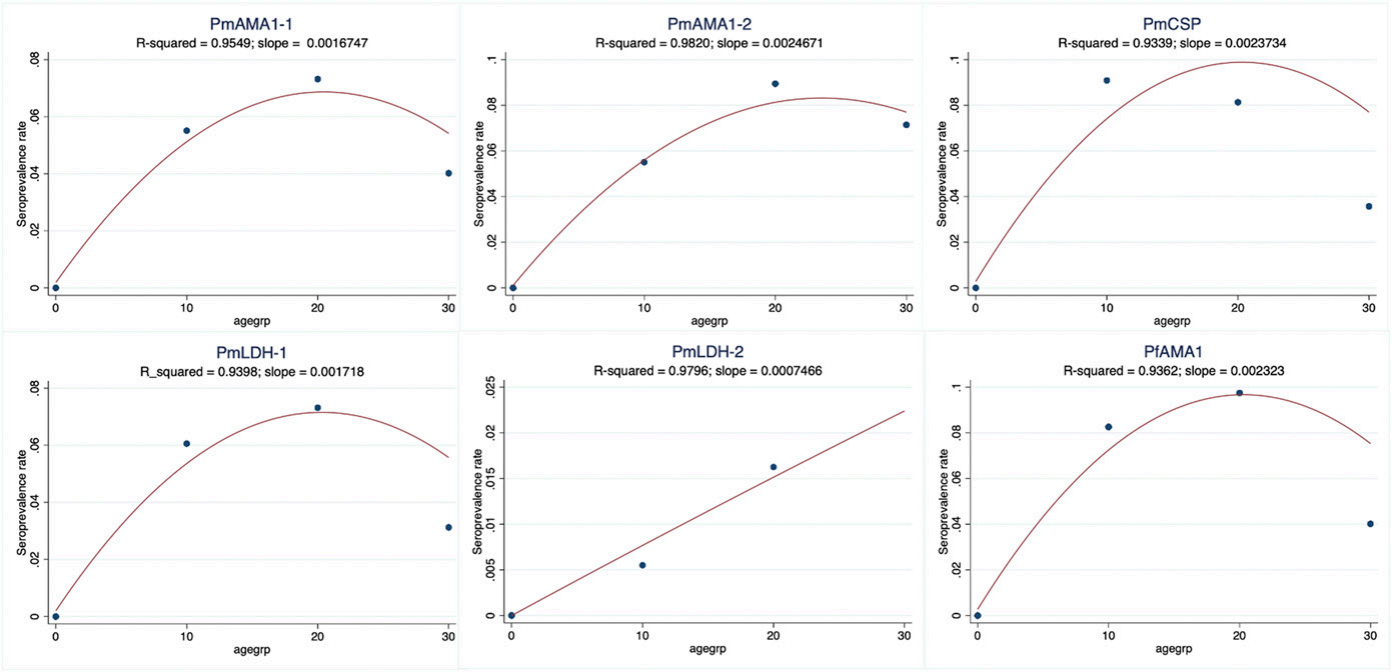
Seroprevalence rates of the different species-specific peptides in different age-groups; the slope is the estimated change of rate over age-group. This figure appears in color at www.ajtmh.org.

Anti-peptide IgG antibody levels were detected against each of the *Plasmodium* spp. peptides designed, and these were relatively higher than the median values of a pool of nonimmune sera, reported as FOC. More than 70% of the samples showed antibody reactivity levels greater than the negative control for each of the peptides (PmAMA1-1 = 73.1%, PmAMA1-2 = 86.7%, *P*. *malariae* CSP [PmCSP] = 78.2%, *P*. *malariae* LDH [PmLDH-1] = 80.9%, PmLDH-2 = 87.2%, and PfAMA1 = 80.3%). From the area under the receiver operator curve for the different peptides, the FOC responses of all the peptides could predict up to 70% of the results obtained by microscopy (range = 62.6–70.5%) and RDTs (range = 66.4–71.9%) (Figure 4). The *P. malariae*–specific anti-peptide antibody reactivities relative to the *P. falciparum* peptide for samples from the first survey expressed significantly higher *P. malariae* responses in Calabar than in Lagos with the exception of the PmCSP peptide, to which Lagos samples were more reactive (Supplemental Figure 1). Because of the small number of samples from Lagos and some of the health facilities in Calabar, all samples were subsequently analyzed together, and the geographical location was not considered. As observed with *P. falciparum*, seroprevalence increased with age for all the *P. malariae* peptides from the age of 10 years (Figure 5). Proportions of seropositivity in younger than 10-year age-groups were variable between antigens, with no consistent pattern between peptides (Supplemental Figure 2). The proportion of seropositivity decreased for PmLDH-1, whereas it increased for PfAMA1 for this younger age-group.

## DISCUSSION

The prevalence of non-*falciparum* species was relatively heterogeneous in health facilities in the southwest and south–south zones of Nigeria, supporting the need for broadening the focus of the National Malaria Control Program beyond *P. falciparum.*^26–29^ Nigeria accounts for an estimated 25% of global malaria cases, mostly considered to be due to *P. falciparum* infection.^30^ With the push for malaria eradication, this indicates that other human *Plasmodium* species should also be considered in current elimination strategies and evaluation of intervention outcomes. Unlike *P. falciparum*, *P. malariae* infections were more prevalent in individuals older than 5 years. Current malaria intervention strategies such as seasonal malaria chemoprevention (SMC) usually target children aged 3–59 months. Hence, older age-groups in *P. malariae*–endemic areas could serve as a reservoir for future expansion of this parasite if *P. falciparum* is selectively eliminated. Higher prevalence of *P. malariae* infections was also observed in older age-groups in Papua New Guinea based on a sensitive molecular diagnostic method, post-PCR ligase detection reaction–fluorescent microsphere assay, compared with light microscopy.^31^ Although overall malaria parasite species differ, the rates for *P. malariae* infections were comparable to those reported for Papua, despite the wide geographic distance and additional prevalence of *P*. *vivax* in Papua. A wider surveillance and reporting of non-*falciparum* malaria parasite species and broadening of future malaria elimination strategies to include all population age-groups at risk of infection with *P. falciparum* and non-*falciparum* species is suggested. Identification of five cases of *P. vivax* and a 10.9% prevalence of *P. malariae* in a recent study of southern Nigerian communities further corroborates our findings and need for enlarged surveys.^29^

Despite the lower prevalence of infection in children < 5 years of age, the odds of *P. malariae* mono-infections were higher in this age-group than in individuals aged > 15 years. This could reflect the level of exposure and immunity, as younger children may be protected by maternal antibodies as well as interventions such as insecticide-treated bed nets and SMC.^32^ The age distribution also mirrors the pattern seen for *P. falciparum*, where an age shift in high infection rates to older children has been reported across Africa following large scale-up of malaria interventions.^33^ The odds of *P. malariae* mono-infection also varied between health facilities, with lowest infection odds in Ifondo, which had, by molecular methods, the highest prevalence of *P. falciparum* infections. This, however, would require further investigation because of the small numbers reported here, reflected in the wide CIs. Nonetheless, some studies have reported higher *P. malariae* prevalence rates when outside the *P. falciparum* peak transmission, suggesting an interaction between the occurrence of these species.^34–36^ Such dynamics could be driven by co-immune mechanisms as the genomes and proteins of these species are highly similar,^37^ hypothetically allowing for high immune responses against *P. falciparum* to eliminate *P. malariae* from coinfections. There could also be an ecological dynamics in which higher multiplication rates and shorter cycle time of *P. falciparum* would displace *P*. *malariae*, which often occurs at low parasite densities and prefer to invade older red blood cells (RBCs).^4^ Infected RBCs are cleared by the spleen during malaria infections,^38,39^ which may in turn remove *P. malariae* from peripheral circulation. However, *P. malariae* has been reported to persist following treatment of mixed species malaria with artemisinin combination therapies,^40–42^ an indication that further studies are needed to elucidate the dynamics and consequences of malaria parasite coinfections. This will require specific surveillance tools, including sensitive and specific diagnostic and serological assays.

In the absence of whole antigen ELISA assays for *P. malariae* and other non-*falciparum* infections, species-specific peptide ELISA assays provided an alternative tool for determining seroprevalence of antibodies against *P*. *malariae*. Predicted peptide epitopes from *P. malariae* AMA1, CSP, and LDH proteins were all recognized by human IgG antibodies, evidence of specific immune responses in exposed communities. Higher relative antibodies were seen in the south–south zone with higher malaria transmission. Thus *P. malariae* elicits specific antibody responses, which could be targeted for the development of sensitive surveillance tools. Seroprevalence has been used as a surrogate for exposure, enabling the determination of force of infection and age-specific immunity for *P. falciparum* and *P. vivax*, in endemic regions.^24,43–45^ ELISA techniques for the detection of antibodies have also been widely used for the diagnosis of infectious diseases and have been used in malaria since the 1970s.^46^ Adequately powered studies to compare and predict transmission intensity of *P*. *malariae* and other non-*falciparum* species across sSA would be useful for strategizing toward control and elimination of all malaria parasite species.

The first survey carried out in 2017 showed a lower prevalence of non-*falciparum* species in Lagos than in Calabar. This was not surprising as urbanization in Lagos metropolitan city, with better housing and access to health services, will limit malaria transmission despite similar vector populations.^47^ A consistent pattern of increased urbanization coincident with decreasing malaria transmission and elimination over the past century has been reported.^48^ Although this study was not designed to determine the epidemiology of *P. malariae*, a properly designed epidemiological study would be more informative. The non-*falciparum* infections in the urban area were submicroscopic and could only be detected by sensitive molecular methods. This emphasizes the challenges to malaria diagnosis with decreased prevalence and more submicroscopic infections. Sensitive and accessible diagnostic tools for routine detection of all human *Plasmodium* species therefore remain important. Microscopy, which is still considered the gold standard for malaria diagnosis, is laborious and time consuming for *Plasmodium* speciation, requiring expert microscopists. Current antigen-based pan-species RDTs also present peculiar challenges, including low sensitivity and lack of species specificity.^49^ The poor sensitivity for pan-species *Plasmodium* detection was recently demonstrated in an experimental human blood-stage model for *P. malariae* infection, where RDTs targeting pan-genus LDH enzyme (pLDH) remained negative despite the presence of symptoms consistent with malaria 72-hour before testing.^50^

Most of the malaria infections detected by PCR were low-grade infections or submicroscopic, which is a well-documented limitation of microscopy, and this may explain the high discrepancy between *Plasmodium* species detected by microscopy compared with PCR. As for *P. falciparum*, ultrasensitive molecular diagnostics that target multi-copy genes will be relevant to assess the true burden of non-*falciparum* malaria infections.

Despite recent reports of *P*. *vivax* detection in Duffy-negative individuals in sSA, including southwestern Nigeria,^51^ no *P*. *vivax* infection was detected. The prevalence of *P*. *vivax* in Duffy-negative individuals could therefore be specific to some susceptible populations driven by yet undetermined biological and environmental factors.

In conclusion, the prevalence rates of non-*falciparum* species reported here indicate that approaches for routine diagnosis in endemic settings should be encouraged to ascertain the true burden of these species as well as for appropriate interventions toward pan-species malaria elimination. Studies to determine the prevalence and dynamics of non-*falciparum* species as well as associated risk factors in endemic populations need to be expanded across sSA.

## Supplemental figures

Supplemental materials
