# Supplementary figures and images for: Seroprevalence and Parasite Rates of Plasmodium malariae in a High Malaria Transmission Setting of Southern Nigeria
Source: Am J Trop Med Hyg. 2020 Oct 26;103(6):2208–16. doi: 10.4269/ajtmh.20-0593 (PMC7695047; doi:10.4269/ajtmh.20-0593)

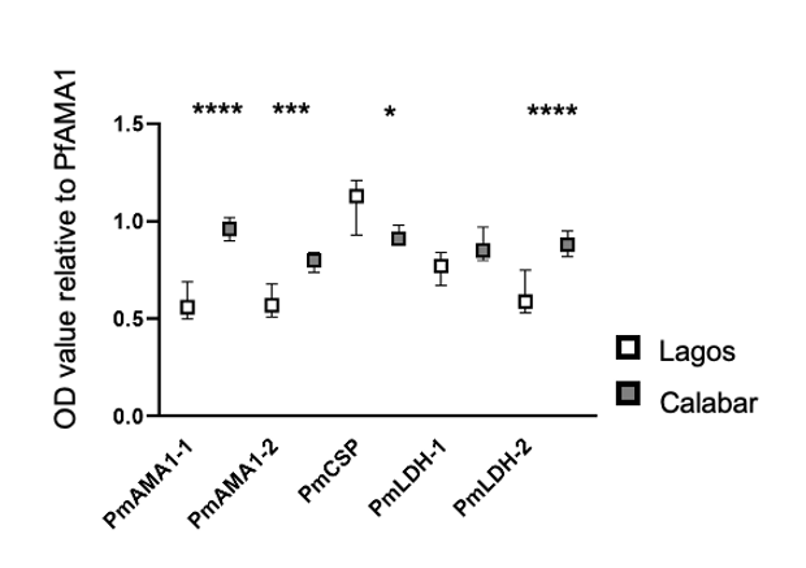

Supplement: Supplementary file 1 [file tpmd200593.SF1.tiff]

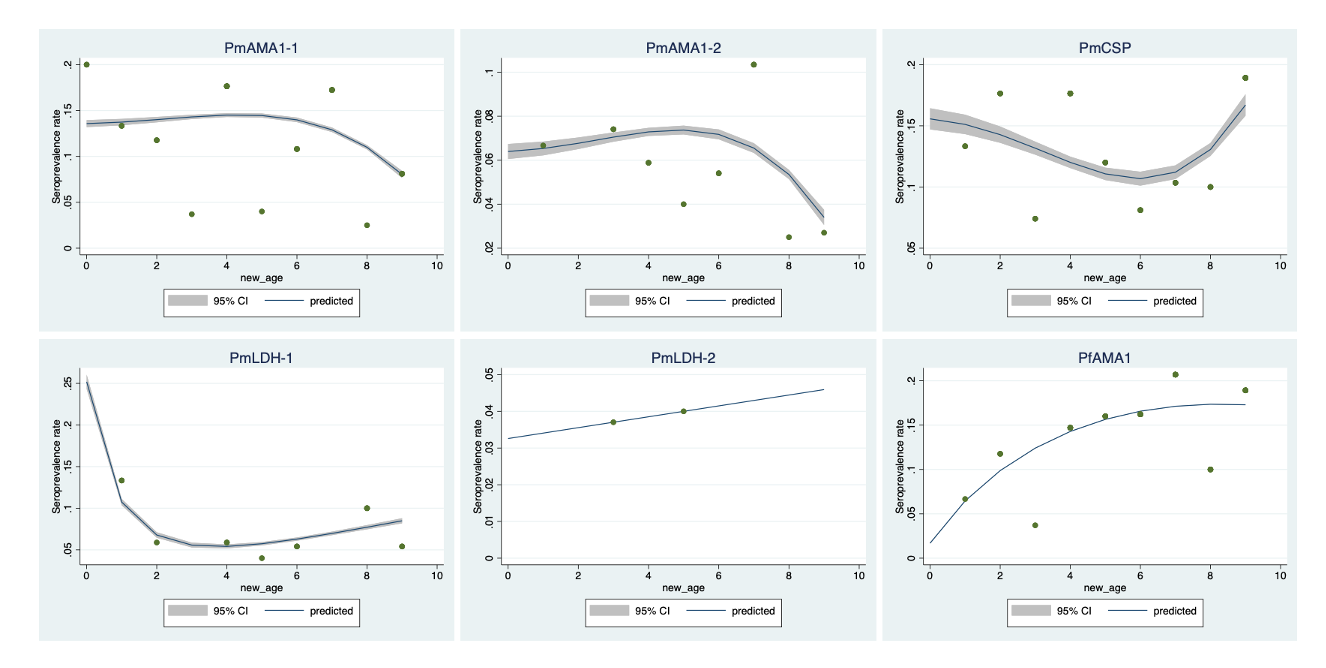

Supplement: Supplementary file 2 [file tpmd200593.SF2.tiff]
